# Characterization of two Mutator insertion alleles of *Chr101* during maize vegetative development

**DOI:** 10.17912/micropub.biology.002431

**Published:** 2026-09-29

**Authors:** Alexia Gulino, Gursimer Anand, Mya Goodson, Navjot Nijjar, Ketzia Obi, Maria Salazar, Dafang Wang

**Affiliations:** 1 Biology, Hofstra University, Hempstead, NY, United States

## Abstract

CHR101 ( *ZmDDM1A* ) encodes a chromatin remodeling factor involved in DNA methylation maintenance and transposable element silencing in maize. We analyzed two *Mutator (Mu)* insertion alleles of *Chr101* : *chr101-m1* , containing an insertion in the 5′ untranslated region, and *chr101-m3* , containing an insertion in exon 3. Quantitative RT-PCR revealed that *Chr101* transcript abundance was reduced by approximately 76-93% in both homozygous mutant lines at juvenile (V4) and adult (V7) vegetative stages. Reduced expression of *Aspartic Proteinase A1* ( *Apa1* ) was also observed in the mutant backgrounds examined.

**Figure 1. Characterization of Chr101 ( ZmDDM1A ) Mutator insertion alleles and transcript accumulation in mutant plants:** **(A)** Gene model of *Chr101* (GRMZM2G177165/Zm00001d007978) showing the locations of the *Mu* insertions. Numbered boxes indicate exons, with dark blue representing coding sequences (CDS) and light blue representing untranslated regions (UTRs). Red arrows indicate the insertion sites of *chr101-m1* and *chr101-m3* . Black arrows indicate primer positions used for genotyping and expression analyses. **(B–E)** Quantitative RT-PCR analysis of *Chr101* and *Aspartic Proteinase A1* ( *Apa1* ) transcript abundance in V4 and V7 leaf tissues. Expression levels in homozygous mutant plants were normalized to *Ubiquitin* and compared with wild-type B73 plants at the corresponding developmental stage. Relative expression values were calculated using the 2^-ΔΔCt method. Three technical replicates were averaged for each biological replicate prior to analysis. Statistical comparisons were performed using Student’s t-test on biological replicate ΔCt values (Yuan et al., 2006). Biological replicate numbers were B73 V4 (n = 2), *chr101-m1* V4 (n = 4), *chr101-m3* V4 (n = 2), B73 V7 (n = 2), *chr101-m1* V7 (n = 4), and *chr101-m3* V7 (n = 2). Error bars indicate ± standard error of the mean. ns, p ≥ 0.05; * p < 0.05; ** p < 0.01; *** p < 0.001.

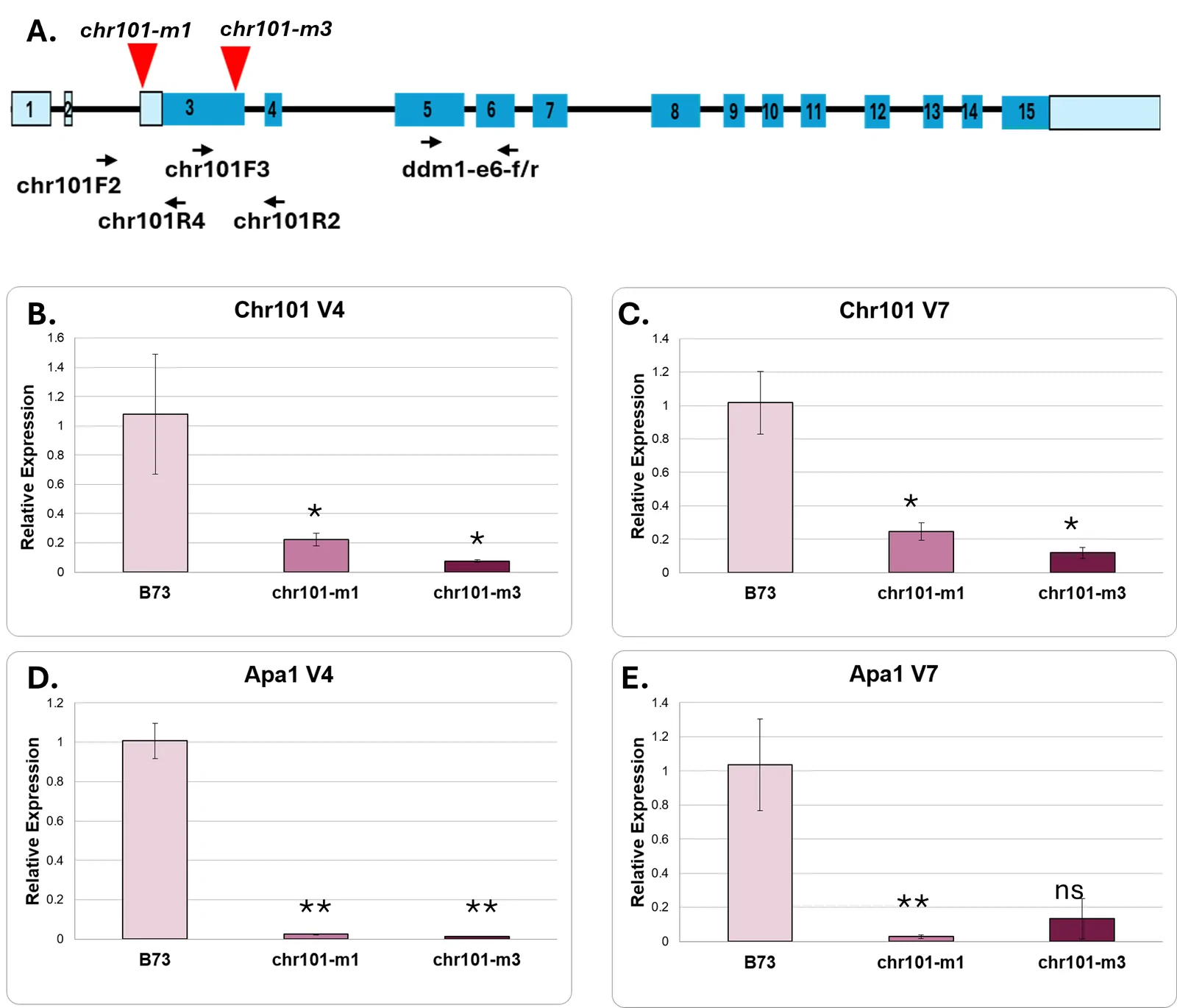



## Description

Transposable elements (TEs) comprise approximately 85% of the maize ( *Zea mays* ) genome and are maintained in a transcriptionally silent state through epigenetic mechanisms including DNA methylation and chromatin remodeling (Schnable et al., 2009; Stitzer et al., 2021; Osakabe et al., 2021). The maize locus *Chr101* ( *ZmDDM1A* ; GRMZM2G177165; Zm00001d007978) encodes an ortholog of Arabidopsis DECREASE IN DNA METHYLATION 1 (DDM1), a chromatin remodeling factor involved in maintaining DNA methylation and transcriptional silencing in nucleosome-dense chromatin (Li et al., 2014; Long et al., 2021). Previous studies demonstrated that disruption of *Chr101* affects DNA methylation and chromatin-associated RNA-directed DNA methylation (RdDM) pathways in maize (Li et al., 2014; Fu et al., 2018; Long et al., 2021).

*Mutator (Mu)* transposable elements are widely used for gene tagging and functional analysis in maize because their insertions frequently disrupt gene expression and can be readily tracked in genetic populations (Robertson, 1978; Settles et al., 2004; McCarty et al., 2005). In this study, we examined two independent *Mu* insertion alleles of *Chr101* . The *chr101-m1* allele contains a *Mu* insertion within the 5′ untranslated region (UTR), whereas *chr101-m3* contains a *Mu* insertion within exon 3 of the coding sequence ( Figure 1A ). These alleles provide an opportunity to compare the effects of 5′ UTR and coding-region insertions on *Chr101* transcript accumulation during vegetative development.

Homozygous mutant lines were utilized for all downstream expression analyses. Zygosity was determined using insertion-specific junction primers and flanking genomic primers and was subsequently verified through sequence alignment of insertion-flanking genomic DNA (Supplementary Figures S1–S2).

Both *Mu* insertion alleles exhibited substantial reductions in *Chr101* transcript accumulation (Supplementary Data File S1). For the 5′ UTR insertion allele, *chr101-m1* , *Chr101* expression was significantly reduced relative to B73 at both the V4 (p = 0.019) and V7 (p = 0.024) developmental stages ( Figure 1B, C). Transcript abundance was reduced by approximately 79% at V4 and 76% at V7. Similarly, the exon 3 insertion allele, *chr101-m3* , showed significantly reduced *Chr101* expression at V4 (p = 0.025) and V7 (p = 0.024), corresponding to reductions of approximately 93% and 88%, respectively ( Figure 1B, C). In both developmental stages, the exon 3 insertion was associated with a larger reduction in transcript abundance than the 5′ UTR insertion, although both alleles produced substantial decreases. These observations indicate that insertions in either the 5’UTR region or coding sequence function as strong transcriptional knockdown alleles of *Chr101* .

Previous work by Stroud and McGinnis (2017) reported reduced *Chr101* transcript abundance in both *chr101-m1* and *chr101-m3* homozygous mutants at the V9 developmental stage. Our results extend these observations by demonstrating that reduced *Chr101* expression is already detectable at V4 and remains evident at V7.

In addition to *Chr101* , expression of *Aspartic Proteinase A1* ( *Apa1* ; Zm00001d044132 or GRMZM2G130333) was examined as a representative euchromatic gene. Based on the B73 RefGen_v5 genome assembly, *Apa1* resides within a gene-dense interval containing eight annotated genes within approximately 100 kb (Jiao et al., 2017) and was not selected based on any previously established relationship with CHR101.

*Apa1* expression was significantly reduced in the *chr101-m1* line at both V4 ( *p* = 0.0084) and V7 ( *p* = 0.0032), corresponding to reductions of approximately 98% and 97%, respectively, relative to B73 controls ( Figure 1D, E). *Apa1* transcript abundance was also significantly reduced in the *chr101-m3* line at V4 ( *p* = 0.0014), corresponding to an approximately 99% reduction relative to B73 ( Figure 1D ). Although mean *Apa1* expression was lower in the *chr101-m3* line at V7, the difference was not statistically significant ( *p* = 0.191). Thus, lower *Apa1* transcript abundance was observed in both *chr101* allele lines at V4 and in *chr101-m1* at V7, although the association was not statistically significant in every comparison.

Although DDM1 proteins are best known for their functions in heterochromatin, maize ZmDDM1 also associates with transcription start sites and other euchromatic regions, and loss of maize DDM1-type remodelers affects RdDM-associated methylation and small-RNA accumulation near genes (Fu et al., 2018; Long et al., 2021). These findings demonstrate that ZmDDM1 is associated with gene-proximal chromatin and provide a possible context for the reduced *Apa1* transcript abundance observed here. The consistent direction of the effect across two independent *chr101* alleles supports the reproducibility of this association. However, *Apa1* has not been identified as a direct ZmDDM1 target, and the mechanism underlying this expression difference remains unresolved.

Overall, these results further characterize two widely used *Chr101* mutant alleles and extend previous observations of reduced *Chr101* expression to the V4 and V7 stages of vegetative development. Both alleles function as strong knockdown alleles at the transcript level. Reduced *Apa1* transcript abundance was also observed in both mutant lines in multiple comparisons, although whether this association represents a direct or indirect consequence of reduced CHR101 activity remains unknown.

## Methods

**Plant Materials and Growth**

The wild-type line B73 was acquired from the Maize Genetics Cooperation Stock Center. Seeds for *chr101-m1* (B73 background) and *chr101-m3* (B73 background) were generously donated by the Springer Lab (University of Minnesota) and were originally described by Li et al. (2014). Premier B10281RG ProMix was used to propagate seeds in small pots. At the V4 developmental stage, seedlings were transferred to larger pots for the remainder of the experiment. Plants were grown under greenhouse conditions at 24–29°C and approximately 80% humidity and were watered twice per week.

**Genotyping**

Leaf tip tissue samples (approximately 2 cm in length) were collected for genotyping. DNA extraction was performed using the Quick-DNA™ Plant/Seed Miniprep Kit (Zymo Research D6020). To confirm homozygosity of the *Mu* insertions in *Chr101* , PCR was performed using PCR Master Mix (Sydlabs, MB067-EQ2B). Reactions utilized flanking and junction primer sequences specific to the *Mu* transposable element. Actin primers were used as a positive control, and nuclease-free water was used as a negative control. Thermal cycling conditions were 95°C for 3 min; 35 cycles of 95°C for 30 s, 60°C for 30 s, and 72°C for 30 s (1 min per kilobase); followed by a final extension at 72°C for 5 min. Amplified products were visualized on 1% agarose gels using a GelDoc Go Imaging System (Bio-Rad).

**RNA Extraction and Quantification**

Leaf tip tissues (~2 cm) from Leaf 4 or Leaf 7 were collected from plants with confirmed homozygous *Mu* insertions at the V4 and V7 developmental stages, respectively. Samples were stored in TRIzol Reagent (Invitrogen) and ground prior to RNA extraction using the Direct-zol™ Miniprep Plus Kit (Zymo Research R2072). RNA quality was assessed using the Qubit™ RNA Broad-Range Assay Kit (Thermo Fisher Scientific), the Qubit™ RNA IQ Assay (Invitrogen), and agarose gel electrophoresis.

**qRT-PCR and Data Analysis**

To measure gene expression, qRT-PCR was performed using the Luna® Universal One-Step RT-qPCR Kit (E3005X; New England Biolabs) on a CFX Connect Real-Time PCR Detection System (Bio-Rad). Transcript levels of *Chr101* , *Apa1* , and *Ubiquitin* (reference gene, GRMZM2G409726) were measured. Relative expression values were calculated using the 2^-ΔΔCt method (Livak and Schmittgen, 2001). Three technical replicates were performed for each biological replicate and were averaged prior to analysis. Biological replicate numbers were B73 V4 (n = 2), *chr101-m1* V4 (n = 4), *chr101-m3* V4 (n = 2), B73 V7 (n = 2), *chr101-m1* V7 (n = 4), and *chr101-m3* V7 (n = 2). Statistical comparisons between genotypes were performed using Student’s t-test on biological replicate ΔCt values following the framework described by Yuan et al. (2006). Relative expression values are reported as means ± standard error of the mean.

## Reagents

**Table:** 

Primer Target	Name	Sequence (5' to 3')
Genotyping PCR		
*chr101-m1* Flank	chr101F2	AAAGCTTCCGTTTCCTTCAGTTCAC
*chr101-m1* Flank	chr101R4	CTCCAGTAGTCGCTCTTCCTCCTTC
*chr101-m3* Flank	chr101F3	GAAGAGGCTGCTAGACTTGCTTTTG
*chr101-m3* Flank	chr101R2	TCTTCTACCTGTGGCTGTTCAGCTTGAG
*Mutator* TE	Museq	CGCCATGGCCTCCATTTCGTCGAATC
RT-qPCR		
*Chr101* Exon 6	ddm1-e6_f	TCATCCACAACAACATACTTCCACT
*Chr101* Exon 6	ddm1-e6_r	TGCCCACAAATGTTGGCCCT
*Apa1*	apa_qf	TGGCGGAAGGAGCACTGGAA
*Apa1*	apa_qr	CCGGCCGCTCCAATCTGTTC
*Ubiquitin*	Ubi-F	GTCATAGTTCTGGGTAGTACGC
*Ubiquitin*	Ubi-R	TGGAGGTTGTCAAAGTATCTGC

## Data Availability

Description: PCR using insertion-specific and flanking primers to confirm the genotypes of plants. Resource Type: Image. DOI: https://doi.org/10.22002/mrvke-e5r18 Description: Sequence alignment between the junction PCR product with Chr101. Resource Type: Image. DOI: https://doi.org/10.22002/4psz8-src28 Description: Raw qRT-PCR data and expression calculations. Resource Type: Dataset. DOI: https://doi.org/10.22002/25qsq-f7989
